# Human Rights Research and Ethics Review: Protecting Individuals or Protecting the State?

**DOI:** 10.1371/journal.pmed.1001325

**Published:** 2012-10-16

**Authors:** Joseph J. Amon, Stefan D. Baral, Chris Beyrer, Nancy Kass

**Affiliations:** 1 Health and Human Rights Division, Human Rights Watch, New York, New York, United States of America; 2 Johns Hopkins Bloomberg School of Public Health, Baltimore, Maryland, United States of America; 3 Center for Public Health and Human Rights, Johns Hopkins University, Baltimore, Maryland, United States of America; 4 Berman Institute of Bioethics, Johns Hopkins University, Baltimore, Maryland, United States of America

## Abstract

Joseph Amon and colleagues discuss the challenges of conducting human rights research in settings where local research ethics committees may favor the interests of the state over the interests of research participants.

**Summary Points**

- Recently there has been a dramatic expansion in research conducted in low- and middle-income countries, as well as research ethics committees (RECs) in these countries.
- RECs in low- and middle-income countries have little experience overseeing human rights research and may be subject to government control or influence that may favor the interests of the state over the interests of individual research participants.
- Many human rights investigators are trained in disciplines with ethical codes and professional norms, but do not typically engage RECs nor see human rights documentation as research, and they tend to view REC approval as counterproductive to the protection of research participants.
- Case studies of human rights research can provide important lessons on navigating conflicts of interest posed by some local (i.e., in country) RECs.
- Expanding the use of community engagement and developing strong ethical operating principles can help ensure that individuals and researchers are protected in human rights research and investigations.

## Background

Human rights violations play an important role as determinants of, or structural barriers to, health [1]–[6]. Research, investigation, and documentation focused on human rights have led to the development of rights-based interventions [7],[8] and the promotion of human rights in the core strategies of international health organizations [9],[10].

At the same time, health and human rights investigations raise complex ethical and methodological challenges [11]. Key questions have emerged about the roles of ethical review and research ethics committees (RECs) when criminalized or marginalized populations are part of research or program efforts [12],[13]. Human rights researchers may also follow ethical codes and professional norms such as those of journalists or lawyers, for example, but these do not typically engage RECs and may in fact define their work differently than biomedical or epidemiologic definitions of “research” [14]–[16]. Furthermore, members of local (i.e., in country) RECs may have conflicts of interest when state actors have a role in or supervision over RECs and can exert their influence to limit the scope of or impede investigations into human rights abuses.

In some circumstances, interests other than ensuring the sound protection of research participants may come to dominate the decisions that RECs make, including whether they agree to review the research and/or allow the research to be conducted at all. Researchers aware of these decision-making processes may “self censor” the focus of their research or choose to conduct research elsewhere. As increasing amounts of research are conducted on the impact of human rights on health, more attention is needed on the roles of RECs and researchers to ensure genuine protection of the individuals involved in human rights investigations.

Here we present examples of how human rights researchers can address complex ethical challenges by building the capacity of community-based organizations representing vulnerable populations and by adopting ethical operating principles. We illustrate our policy proposals using case studies of research involving men who have sex with men (MSM) in Africa, ethnic minorities in Myanmar, and individuals in compulsory drug treatment centers in Asia.

## Human Participant Protections

The protection of participants in health-related research has evolved into a well-articulated international framework supported by normative documents, conventions, and, in growing numbers of jurisdictions, laws. Key among these are the World Medical Association's 1964 *Declaration of Helsinki* [17], the US Department of Health and Human Services *Belmont Report* and regulations for the protection of research participants [18],[19], the Council for International Organizations of Medical Sciences international ethical guidelines [20], and the International Conference of Harmonisation of Technical Requirements for Registration of Pharmaceuticals for Human Use (http://www.ich.org/). All of these guidelines require prior review of research by an REC before research can be implemented. More recently, the World Health Organization published standards for such committees outlining key requirements for their structure, governance, and review standards [21].

Over the last ten years, there has been extraordinary growth in the numbers of RECs in low- and middle-income countries (LMICs). As new committees in LMICs have emerged, many countries have adopted a structure whereby local committees, affiliated with specific research institutions or organizations, are supported by a national committee. The national committee is in charge of creating policies, providing oversight, and, in some cases, performing an additional, final review.

Unfortunately, the methodology and intent of human rights research has not been fully considered in existing standards and guidelines on the ethical conduct of research. Similarly, RECs have traditionally been orientated to biomedical and epidemiologic research and have rarely considered human rights research. While principles such as autonomy, beneficence, non-malevolence, and justice are common to ethical codes in diverse disciplines [14]–[16],[18],[22], the definition of “research” and the requirement for REC review are not universal across different types of research.

## Defining “Research”

The definition of research and the difference between health research (typically requiring ethics review) and monitoring, evaluation, or practice (typically exempt from review) are not straightforward [23]. For individuals engaged in rights research and RECs considering their jurisdiction over such research, the determination of whether a human rights investigation constitutes research can be contentious and may reflect differences in disciplinary training and professional norms.

Health and human rights investigations can often be considered “non-research” under the US Department of Health and Human Services and international definitions that define research as developing “generalizable knowledge” [19],[20]. Documentation of particular human rights abuses, factors that contribute to particular cases of human rights abuse, or human rights protections in particular situations are not usually considered “generalizable.” While broader surveys determining the prevalence of abuses may be considered research, in some cases they may be considered monitoring, which, again, is commonly exempt from review. In addition, individuals who provide testimony or evidence of human rights abuses are not traditional research participants. Instead, these individuals have an important motivation for engagement in human rights investigations, that is, for seeing such investigations as perhaps their only means of achieving justice for themselves and their communities. Thus, their view of the balance of “risk” versus “benefit” may be substantially different from the view held by biomedical researchers or REC members.

## Conducting Research on MSM and HIV in Africa

Recently identified HIV outbreaks among MSM in several African countries have revealed many neglected or hidden human rights abuses. These abuses include discrimination in access to HIV prevention and treatment, lack of access to justice, police abuse, arbitrary arrest and detention, and ill-treatment and torture. In nearly all African countries in which research has been conducted, HIV infection rates have been markedly higher among MSM than among other men of reproductive age [24]–[29]. These epidemics are occurring among largely hidden, stigmatized, and—in many countries—criminalized MSM communities, challenging research and service provision [30].

In some countries, police have specifically targeted outreach workers providing information and condoms to MSM [31],[32], and health-care workers have been complicit in efforts to “prove” homosexuality with forced anal exams [33]–[35]. In Uganda, conducting research on MSM, including investigations of possible human rights abuses, has become difficult or impossible. Reasons for this difficulty include proposed legislation to make sodomy a capital offense and to criminalize the failure to report individuals suspected of engaging in homosexual behaviors, and targeted violence against individuals identified as MSM, including murder [36],[37].

Nevertheless, MSM health service providers and gay service and rights organizations and activists in many African countries have been enthusiastic partners in HIV-related programs, including research, even though governments have been reluctant to support research on MSM. In several cases, governments have actively opposed research that would lend credence to the reality that lesbian, gay, bisexual, and transgender persons exist in their countries, and to the fact that MSM are at elevated risk for HIV infection [37],[38].

At one site of a multi-country study being conducted by two of the authors of this article (S. D. B. and C. B.), the head of the only university-based REC informed the research team that, since homosexuality was criminalized in the country, no research protocols related to MSM would be accepted for review. The REC chair told the researchers that the role of the REC included the protection of social and cultural values of the country. While RECs may legitimately reference social and cultural values in considering what constitutes risk to individual human participants, the REC in this case defined its role well beyond protection of human welfare to instead reinforce a political position of the state.

In response, researchers engaged community-based organizations serving MSM in the country to gauge the level of support for the study, and trained community leaders on research ethics [39]. The study protocol was then reviewed by community leaders, who suggested protocol changes based on further community consultation. At the same time, the protocols were also reviewed by a REC in the US that was informed that the in-country REC had refused to review the protocol. After approval by the US REC, the researchers decided that the final decision to proceed should be made by the community-based organizations in country based upon their assessment of the risks and benefits of the research. Community members also participated in validating research findings, and members of the community presented the results to their peers and in domestic and international forums.

## Investigating Health and Human Rights in Myanmar

In democratic societies where government legitimacy has broad acceptance, and where ministries of health are seen as working to advance the health and well-being of the population, researchers rarely question whether academic or state entities have the right to form and oversee RECs. In contrast, in repressive societies, and where an REC is seen as not representative of, or legitimately protecting the interests of, a particular vulnerable group (e.g., prisoners, women, or an ethnic or religious minority), RECs may be understood as agents of the state: prioritizing the protection of state interests over those of research participants.

In the case of Myanmar, decades of civil and ethnic conflict have left large areas of the country under contested political control. Several major ethnic nationalities, including the Karen, Kachin, Chin, Shan, Mon, and Wa, have been in open armed conflict with the ruling military-backed regime or have cease-fire agreements that allow them considerable autonomy. Most of these ethnic groups do not have formal relationships with the ruling government.

In working with ethnic populations in border zones since 1992, we (as well as collaborators from the University of California, Los Angeles, and other entities) have struggled with the question of who most legitimately represents these populations and specifically who should safeguard their rights and interests if researchers or investigators want to collect data. For individuals with no formal communication with the regime they are fighting, the concept that this regime could make decisions for their health and well-being is both absurd and offensive. However, popular support for the government in exile is strong among most of Myamnar's ethnic national organizations, and this exiled government has a well-established health and welfare committee. Consequently, we have helped to establish and build the capacity of an REC composed of Myanmar physicians and nurses in exile, community health workers, community members, and faith-based leaders. This group has now had several years of experience functioning as an REC and reviewing proposals, and their authority has been accepted by RECs at US institutions [40]–[42].

## Documenting Abuses in Compulsory Drug Treatment Centers

Between July 2007 and September 2011, Human Rights Watch conducted investigations of compulsory detention of drug users in China [43],[44], Cambodia [45], Viet Nam [46], and the Lao People's Democratic Republic [47]. In these countries, drug use is legal but drug users are subject to extrajudicial administrative detention for the purpose of compulsory treatment of drug dependency. The investigations conducted by Human Rights Watch included interviews with individuals recently detained in drug detention centers; key informant interviews with non-government organizations, funding entities, and, in some cases, government officials; review of relevant government laws and policies; and review of international donor policies and programs in drug detention centers. The investigators found that individuals in drug detention centers were routinely held without clinical determination of drug dependency or due process, and once detained were denied evidence-based drug treatment as well as other basic health services. Drug users were often forced to perform arduous physical exercise, military drills, or forced labor, and were subject to physical and sexual abuse.

While research on drug addiction, HIV virology, HIV prevalence, and HIV prevention has been routinely conducted inside detention centers with the approval of government-affiliated RECs and the authorization of the government-controlled detention centers, the specific ethical concerns of conducting research in institutions that violate due process protections have not been addressed. At a minimum, researchers should be expected to accurately characterize the research setting and status of participants. Yet, researchers have often ignored the conditions within and lack of judicial oversight of such centers, presenting them as legitimate treatment facilities [48],[49]. Researchers rarely report on the availability of evidence-based drug dependency treatment [48],[50]–[55] and have obscured the status of research participants (e.g., referring to detainees as “patients” [48] or vaguely alluding to their “complex legal needs” [55]). Published papers also often omit mention of the challenges of conducting independent research [48],[50]–[55]. One study acknowledged using detention center staff to witness consent [55], potentially increasing the risk of coercion. Researchers who do not have full, independent, or ongoing access to detention centers may be unable to assess negative consequences for research participants, and detainees who do not have access to legal counsel or the right to free speech may be unable to file a complaint alleging abusive research.

In response to these challenges, we chose to conduct research with individuals in the community who had been recently released from detention centers. However, human rights monitoring by independent international organizations is not allowed in China, Viet Nam, or the Lao People's Democratic Republic, and we did not feel that local RECs would approve research related to torture and ill-treatment. Therefore, a decision was made to proceed without local REC approval in order to protect both research participants and researchers, who we feared could be targeted by the state for proposing research that is viewed as sensitive to state security or disruptive of government goals of “social harmony.” In place of local REC approval, and because we felt that there was no defined community of recently released drug users to formally consult with (and that community engagement in the context of ongoing persecution would not be safe regardless), researchers developed and followed specific ethical operating principles. In contrast to the typical approach of RECs, where review is limited to the research protocol, every step of the research, from the protocol review to implementation to dissemination of results to scientific, diplomatic, and media audiences included internal ethics review by technical and legal experts.

## Mitigating Risks in Human Rights Investigations

To address the possible conflicts investigators may face in protecting participants in the course of health and human rights investigations, local RECs are needed that can be considered truly independent. In addition, two distinct and complementary strategies—community-based review and the development of strong ethical operating principles—can help protect investigators and participants in health-related human rights research.

In the context of governments that persecute specific populations, actively limit free speech, and routinely punish criticism of the state, RECs are unlikely to be independent. Under these circumstances, using local RECs to safeguard the rights and interests of research participants may be counterproductive, putting both investigators and participants at risk. In these settings, researchers may need to actively engage communities and follow clear ethical operating principles in place of local REC review.

Community-based review and participatory research have a long history and were developed to address community members' concerns about neglect by and communities' mistrust of researchers, health-care systems, and government [56],[57]. Conducted correctly, community-based participatory research (including financial and technical support for community engagement and leadership) creates bridges between policy-makers, scientists, and communities; facilitates reciprocal learning; assists in the development of culturally appropriate measurement instruments and interventions; and establishes a level of trust that enhances both the quantity and the quality of data collected and programs delivered [39],[57]–[61]. While there is a well-established body of literature on engagement of marginalized populations in high-income settings and on some vulnerable populations in LMICs [57],[58], the issues faced by criminalized and violently stigmatized populations have less often been addressed.

One challenge of community-based review is that in many settings the “community” is not homogenous, organized, or able to participate in extensive consultation and review of proposed research. Research with migrants, prisoners, drug users, and criminalized populations is often conducted without a representative advocacy group. In other settings, it may not be clear who legitimately speaks for marginalized populations. In all settings, community-based review can be time-consuming and resource intensive.

In conducting human rights research, particularly in settings where safety may be of particular concern, a critical first step is to have standing procedures on investigator and participant protection. All Human Rights Watch staff who conduct interviews, for example, undergo security training and training on participants' protection and data safety. Researchers can also receive specialized training on how to sensitively interview people in such a way as to minimize risk of re-traumatization, including training on interviewing victims of sexual violence, children, persons in extreme pain, prisoners, and the mentally disabled. All researchers must participate in a security meeting prior to a research mission that establishes chains of communication so that security emergencies can be identified and handled once the mission is in progress. Post-mission meetings are held if security concerns arise, and the security of participants stemming from contact with researchers is monitored. Prior to publication of any findings from research (in the form of reports, journal articles, press releases, opinion pieces, photography, or other media), legal review is required and provides further assessment of research participant protection.

## Conclusion

For individuals who experience human rights abuses, the consequences of reporting that abuse are often uncertain. Yet even when the risk of retaliation is judged to be high, many individuals may be willing to take such a risk in order to press for justice, despite the fact that justice may take years or even decades to be served. Individuals who are a part of communities that are systematically discriminated against, stigmatized, or criminalized may experience high levels of ongoing harm, and see participation in a human rights investigation as one of few means of challenging those abuses or demanding redress.

In the decade to come, RECs in LMICs will likely acquire increasing jurisdiction, resources, and authority over local research. These changes will offer a promise of greater protection for research participants who in the past have faced abuses with little opportunity for redress. But RECs may have little experience in evaluating the inherent risks faced by individuals vulnerable to human rights abuses as well as the risks and benefits from participation in a human rights investigation. RECs, which primarily review pre-research protocols, may also be poorly suited to the review of dynamic investigations using open-ended research methodologies where the risk to participants is less a result of research processes (e.g., questionnaires) than from post-research products (e.g., reports, legal processes, and media coverage).

The use of RECs to limit health and human rights research for political, cultural, or other considerations is a misuse of the legitimate functions of RECs. Careful attention must be paid when local committees assert that their views represent local cultural norms, or that human rights are an illegitimate focus of research as they express foreign values. A critical distinction for researchers is understanding the difference between respecting cultural traditions that are “matters of etiquette, ritual, or religion,” with little or no relation to ethics, from those cultural traditions with ethical (or human rights) implications, such as female genital mutilation or infanticide [62]. Cultural practices or government policies that either deliberately or incidentally serve to suppress or threaten the rights of certain people cannot be respected. RECs, charged specifically with upholding the rights and protection of individuals, should not use culture or “values” as a means to deny human rights.

Increasing attention to human rights as a determinant of health will result in increasing requests to RECs to review research that investigates the role and complicity of state actors, government laws and policies, and social or cultural norms as they relate to health. Stronger, independent RECs trained in human rights may be better equipped to more adequately review this research. When RECs are unable to do so, or where research on human rights or criminalized or marginalized populations is expressly prohibited, researchers may need to rely upon alternative strategies, including engaging communities and following ethical operating principles, to ensure that research participants are protected and that research is ethically conducted. While such innovations do not eliminate all risks, and may be costly in terms of time and resources, the alternatives, which may include acceding to censorship or not conducting investigations at all, are unacceptable limits.

## Abbreviations

LMICs: low- and middle-income countries
MSM: men who have sex with men
REC: research ethics committee
